# Outcome measures in asthma attack trials: can't see the good for the wheeze

**DOI:** 10.1183/23120541.01036-2023

**Published:** 2024-02-26

**Authors:** Richard J. Russell

**Affiliations:** Department of Respiratory Sciences, University of Leicester, Biomedical Research Centre – Respiratory, Glenfield Hospital, Leicester, UK

## Abstract

Asthma is estimated to affect around 262 million people worldwide and prevalence continues to increase [1]. A significant proportion of the burden associated with asthma, for both patients and healthcare systems, relates to exacerbation events, or “asthma attacks” [2]. These vary greatly in severity and are therefore managed in a wide range of settings, including emergency departments, hospital inpatient wards, primary care and the patient's own home. Exacerbations carry a risk of significant harm and even death to the person affected, and impact on their quality of life, activities of daily living and work or school productivity during the recovery phase [3]. It is therefore imperative that exacerbations are treated with the most optimal care possible.

## Shareable abstract

**A systematic review highlights limitations and variability in outcome measures for trials in asthma attack treatment. Now is the time to adopt a consistent approach to ensure best patient care in a precision medicine future.** https://bit.ly/3O4zDHY

Asthma is estimated to affect around 262 million people worldwide and prevalence continues to increase [1]. A significant proportion of the burden associated with asthma, for both patients and healthcare systems, relates to exacerbation events, or “asthma attacks” [2]. These vary greatly in severity and are therefore managed in a wide range of settings, including emergency departments, hospital inpatient wards, primary care and the patient's own home. Exacerbations carry a risk of significant harm and even death to the person affected, and impact on their quality of life, activities of daily living and work or school productivity during the recovery phase [3]. It is therefore imperative that exacerbations are treated with the most optimal care possible.

Ensuring that patients with asthma exacerbations receive the best care across the spectrum of healthcare settings is dependent on good-quality evidence to guide clinician treatment choices. However, the delivery of research trials in high-acuity and time-deprived healthcare settings, such as emergency departments, medical wards and primary care, brings significant challenges. As a result, this arena of research is often neglected compared to studies involving stable patients, which can be delivered in a less pressured and more controlled environment.

One such challenge to research in the acute setting is determining the most appropriate outcome measures. Outcome priorities may vary at different points in a patient's acute treatment journey. For example, in a patient presenting with features of a life-threatening asthma exacerbation to the emergency department, the most important outcome measures for an intervention at that point are likely to be the rapid correction of abnormal physiological parameters such as oxygen saturation. Whereas once a patient is admitted to a medical ward, the most important outcome measure for an intervention may shift to being the duration of the hospital admission or the prevention of future relapse.

In addition, individuals with asthma may place different degrees of importance on different outcome measures, such as admission avoidance or improvement in quality-of-life scores. These priorities may not align with the those of clinicians, researchers or healthcare funders, as has been observed with outcome priorities for novel maintenance treatments in asthma [4, 5].

Finally, not all outcome measures are easily deliverable in the acute setting. For example, spirometry in the early stages of an asthma exacerbation may be difficult for the patient, or there may be a lack of spirometry equipment and trained staff in order to collect this data, particularly “out of hours”. It is easy to see, therefore, why trials in asthma exacerbation may use a mixed bag of study outcomes.

As a consequence of this, the evidence base underpinning the treatments for asthma exacerbation has more limitations than might be expected. Systemic corticosteroids have a robust body of evidence supporting their use in acute asthma exacerbation and are rightly positioned prominently in treatment guidelines [6, 7]. However, once you start asking specific questions about particular outcomes, some evidence limitations start to become apparent. For example, a Cochrane review reporting the risk of relapse after treatment with corticosteroids for acute asthma only identified six studies, totalling 374 patients [8]. Similarly, a 2016 review was unable to make any recommendations about different doses and durations of acute corticosteroid courses, or whether tapering corticosteroids after exacerbation is beneficial, due in part to variability in the outcome measures reported in the included studies [9].

In this issue of *ERJ Open Research*, Howell *et al.* [10] report the findings of their systematic review investigating the outcome measures reported in clinical trials of interventions for treating asthma exacerbations. The authors cast a wide net with their search, by including studies spanning a 50-year period, and using broad inclusion criteria to capture studies from any setting reporting any intervention for the treatment of asthma exacerbation, including pharmacological and ventilation strategies, and patient education. Despite this, only 208 randomised controlled studies were identified, highlighting the surprising paucity of high-quality evidence in this important area.

Howell *et al.* [10] categorised outcomes from the included studies into three broad categories: physiological, healthcare utilisation and patient-reported. They find that the outcomes most often reported in clinical trials are physiological and relate to measures of lung function, with either forced expiratory volume in 1 s or peak expiratory flow rate reported in 95% of the included studies (68% as the primary outcome). Median time from intervention to assessment was only 3 h. The significance of short-term changes in airway physiology to the patient's overall recovery is debatable. While certainly an important component to address in stabilising the acutely unwell patient, bronchoconstriction is highly variable during an asthma exacerbation, with significant fluctuations over hours and days, as well as in response to short-acting bronchodilator medications [11]. Lung function does not relate well to other measures of asthma control in nonexacerbating patients [12], and whether lung function response in the first 3 h of an exacerbation is important to ongoing recovery, or in preventing future relapse, is unclear.

Outcome measures related to healthcare utilisation and treatment failure may be more relevant to the patient's overall recovery. This may include evaluating the requirement for treatment escalation during the acute exacerbation or repeat attendance to a healthcare provider during the recovery phase. These measures lend themselves readily to assessment over a longer follow-up period and may be more important to patients trying to navigate their ongoing recovery around other life commitments. However, Howell *et al.* [10] find that these outcomes are not only reported less frequently but are often poorly defined with inconsistent criteria applied between studies. This variability means that meta-analysis of these outcomes may not be feasible, hampering attempts to combine data and strengthen conclusions. Howell *et al.* [10] also describe similar between-study variability in the way that patient-reported outcome measures are utilised, and find that a wide range of validated and unvalidated assessments are used.

Overall, the findings of Howell *et al.* [10] highlight some of the evidence limitations faced in determining optimal management of asthma exacerbations, particularly in a personalised or precision medicine era. Studies use a broad range of outcome measures, which are often poorly defined, and the timeframes over which these are assessed are often very short. Due to this, comparison of efficacy between different interventions and regarding specific patient-focussed outcomes is difficult. Potential nuances that could be integrated into more optimised treatment guidelines may be missed. Somewhat reassuringly, Howell *et al.* [10] found an increasing trend over time towards larger, adequately powered studies with defined primary outcomes, and greater inclusion of healthcare utilisation and patient-reported outcomes. Therefore, perhaps now is the ideal time to seek to resolve these issues.

Howell *et al.* [10] propose that a more consistent and reasoned approach to the choice of trial outcomes and assessment timepoints is taken. This should include input from patients, to ensure outcomes are reflective of patient priorities, in addition to those of clinicians and healthcare funders. Recent asthma exacerbation is a strong predictive risk factor for further exacerbation [13, 14]. Hence, during the treatment of one exacerbation event, it follows that an important outcome to consider is the effect on future exacerbation risk.

Once a key outcome set is defined, the evidence for existing asthma exacerbation treatments should be reviewed. Where gaps persist, further studies reporting validated clinically important outcome measures should be considered to ensure these are addressed. Consistency of outcome measures across treatment interventions could then facilitate a more detailed evaluation of current clinical guideline recommendations, more accurate comparison between interventions regarding their magnitude of benefit and further optimisation of care pathways. It would also provide reference points against which future novel therapeutics can be benchmarked, allowing easier evaluation of how these should fit into clinical practice.

The COVID-19 pandemic has shown us that well-designed acute intervention studies with clinically meaningful outcomes can be delivered at scale [15], and this is a growing field in a wide range of acute medical problems. It is too easy to assume that existing guidelines are already perfected and not to question the *status quo*. There should be a continuous drive to optimise management, and perhaps shift to a more personalised medicine approach even in the acute phase of an asthma attack. To do this requires strong and comparable data for multiple interventions. Consistent, robust and clinically relevant outcome measures are key to this.
